# Supplementary material for: Femtosecond exciton dynamics in WSe2 optical waveguides
Source: Nat Commun. 2020 Jul 16;11:3567. doi: 10.1038/s41467-020-17335-w (PMC7367278; doi:10.1038/s41467-020-17335-w)
Supplement: Supplementary file 1 — Supplementary Information [file 41467_2020_17335_MOESM1_ESM.pdf]

Supplementary information

Femtosecond exciton dynamics in WSe_2 optical waveguides

Aaron J. Sternbach[1*], Simone Latini[2], Sanghoon Chae[3], Hannes Hübener[2], Umberto De Giovannini[2], Yinming Shao[1], Lin Xiong[1], Zhiyuan Sun[1], Norman Shi[1], Peter Kissin[4], Guang-Xin Ni[1], Daniel Rhodes[3], Brian Kim[3], Nanfang Yu[1], Andrew J. Millis[1], Michael M. Fogler[4], Peter J. Schuck[3], Michal Lipson[5], X.-Y. Zhu[6], James Hone[3], Richard D. Averitt[4], Angel Rubio[2,7], and D. N. Basov[1]

[1] *Department of Physics, Columbia University, New York, New York 10027, USA*
[2] *Max Planck Institute for the Structure and Dynamics of Matter, Luruper Chaussee 149, 22761 Hamburg, Germany*
[3] *Department of Mechanical Engineering, Columbia University, New York, New York 10027, USA*
[4] *Department of Physics, University of California, San Diego, La Jolla 92093, CA, USA*
[5] *Department of Electrical Engineering, Columbia University, New York, New York 10027, USA*
[6] *Department of Chemical Engineering, Columbia University, New York, New York 10027, USA*
[7] *Center for Computational Quantum Physics (CCQ), Flatiron Institute, 162 Fifth Avenue, New York, New York 10010, USA*

* Corresponding author: as5049@columbia.edu

Supplementary Note 1: "Modeling the near-field amplitude"

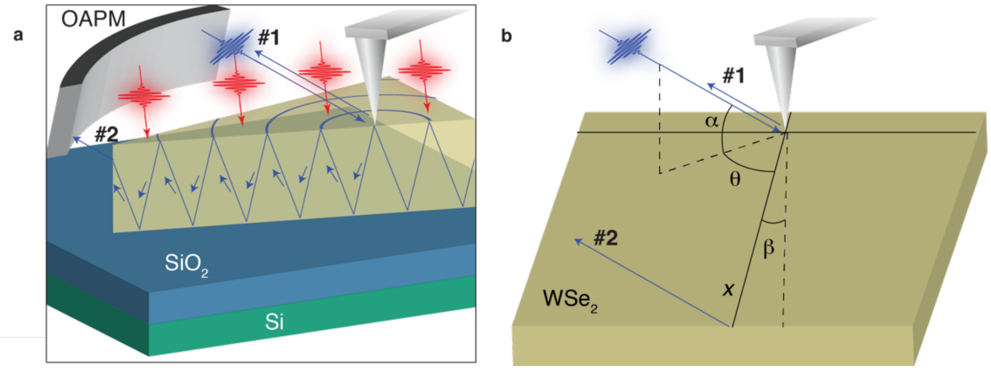

Supplementary Figure 1| a, Schematic of the experiment. Silver tip represents the AFM with a radius of ~20 nm at the apex. Yellow triangular slab denotes the WSe_2 crystal. Red and purple pulses indicate the pump and probe radiation, respectively. **b**, Geometry of the experiment. The incoming probe light is indicated with the purple pulse. Two possible beam paths for the scattered light, labeled #1 and #2 are indicated with purple arrows.

It is instructive to, first, consider the case that a monochromatic continuous wavelength (CW) light source with frequency, $\omega = \omega_0$, interacts with the tip/sample system[1]. Subtleties imposed by broadband sources, which are necessarily employed in time-resolved experiments, are discussed in Supplementary Note 12. In our experiments we illuminate a large area surrounding the AFM tip with pump and probe radiation (Supplementary Fig. 1). Optical contrasts from the near-field are isolated from background radiation using the time-resolved psuedoheterodyne technique[2]. At equilibrium the laser beam #1, with the complex amplitude $E_{1,r}$, is backscattered from the AFM tip. Simultaneously, a photonic waveguide mode is launched with the complex wavevector, $q_r = q_{1,r} + iq_{2,r}$ (see Section 3) and travels within the WSe_2 crystal[3, 4, 5]. The beam following path #2 is scattered from the edge of the WSe_2 crystal. Both of the scattered beams, #1 and #2, reach our detector. We consider the complex electric field amplitude at the position *x* relative to the location of the AFM tip:

$$E_{k,r}(x,t) = C_{k,r}(x) \exp[-q_{2,r}x] \exp[i(q_{1,r}x - \omega_0 t)] \qquad (1)$$

The subscript k is used to indicate the beam path, k = 1, or 2. Beam path #1 (k = 1) is scattered from the AFM probe at x = 0. The waveguide mode, which travels along beam path #2 (k = 2), is also scattered to the detector after traveling a distance, of about *x*, within the WSe_2 waveguide. The near-field amplitude that is detected in our experiments can be written as[5]:

$$S_r(x) \propto \mathrm{abs}[E_{2,r}(x) + E_{1,r}(0)] \ (x>0) \qquad (2)$$

The waveguide mode propagates away from the AFM probe and decays as $C_{2,\mathrm{r}}(x) = C_2/\sqrt{x}$. We assume that the amplitude of the waveguide mode is small, $C_{2,\mathrm{r}} \ll C_{1,\mathrm{r}}$. Indeed, the efficiency of launching the waveguide modes is low and the intensity decays with increasing distance[6]. Then it is straightforward to expand Supplementary Equation 2 as:

$$S_\mathrm{r} \cong \frac{C_2}{\sqrt{x}} \exp\left[-q_{2,\mathrm{r}}x\right] \cos\left(q_{1,\mathrm{r}}x + \varphi\right) + C_{1,\mathrm{r}} \quad (3)$$

In our experiments, photoexcitation is utilized to perturb the properties of a WSe_2 crystal. The pump beam has an elliptical cross-section with the calculated semi-minor and semi-major axes of 9 μm and 16 μm respectively. Thus, the pump intensity is nearly homogeneous over the sub-10 μm fields of view investigated in our work. The pump beam is, furthermore, chosen to have horizontal polarization, which couples weakly to the AFM probe resulting in minimal distortion of the electric field at the tip apex[7]. In our modeling we assume that the coefficient for the amplitude, $C_{2,\mathrm{r}} = C_{2,\mathrm{p}} = C_2$, and phase factor, φ are unchanged by photo-excitation, while the wavevector is modified:

$$S_\mathrm{p} \cong \frac{C_2}{\sqrt{x}} \exp\left[-q_{2,\mathrm{p}}x\right] \cos\left(q_{1,\mathrm{p}}x + \varphi\right) + C_{1,\mathrm{p}} \quad (4)$$

The pump-beam operates at half of the repetition rate of the probe beam (approximately 375 kHz) so that each pair of probe pulses contains a pump ON event, Supplementary Equation (4), and pump OFF event, Supplementary Equation (3). By using two separate digital boxcars to collect the intensity from both of these probe pulses, as described in Ref. [14], S_r and S_p are simultaneously measured at each pixel. The pump-induced change in near-field amplitude is readily calculated:

$$\Delta S = S_\mathrm{p} - S_\mathrm{r} = \frac{C_2}{\sqrt{x}}\left(\exp\left[-q_{2,\mathrm{p}}x\right] \cos\left(q_{1,\mathrm{p}}x + \varphi\right) - \exp\left[-q_{2,\mathrm{r}}x\right] \cos\left(q_{1,\mathrm{r}}x + \varphi\right)\right) - \delta c \quad (5)$$

where the change in near-field amplitude in the interior of the sample is given by $\delta c = C_{1,\mathrm{p}} - C_{1,\mathrm{r}}$. In Supplementary Figures 4, 6 and 7 we display ΔS in units of percent by further dividing ΔS as expressed in Supplementary Equation 5 by the mean-value of the near-field amplitude in the interior of the film $\langle S_\mathrm{r} \rangle$. We re-write the complex wavevector under photoexcitation as $q_{\mathrm{i,p}} = q_{\mathrm{i,r}} + \delta q_\mathrm{i}$ to highlight the pump-induced change to the complex wavevector, δq_i. The subscript i is used to indicate the real or imaginary component of the complex wavevector, i = 1, or 2 respectively. In the specific case that the pump-induced change to the complex wavevector is small, $\delta q_\mathrm{i} X \ll 1$ we may expand Supplementary Equation 5. Where X is the largest distance from the edge of WSe_2 where we investigate the waveguide mode. X ranges from 2-10 μm in our work. If this signal is normalized such that C_2=1 it can be easily verified that to the leading order:

$$\begin{aligned}\Delta S + \delta c &\cong \sqrt{x}\exp\left[-q_{2,\mathrm{r}}x\right]\left(\delta q_1 \sin\left(q_{1,\mathrm{r}}x + \varphi\right) - \delta q_2 \cos\left(q_{1,\mathrm{r}}x + \varphi\right)\right) \\ &= \sqrt{x}\exp\left[-q_{2,\mathrm{r}}x\right] \sqrt{\delta q_1^2 + \delta q_2^2} \cos\left(q_{1,\mathrm{r}}x + \varphi + y\right) \quad (6)\end{aligned}$$

with

$$y = \tan^{-1}\left(\frac{\delta q_1}{\delta q_2}\right) \qquad (7)$$

We emphasize that φ, $q_{1,r}$, and $q_{2,r}$ can be determined from the measurement of S_r in equilibrium. Two unknown quantities remain to describe the line profiles of ΔS: the amplitude of the oscillations, $A \propto \sqrt{\delta q_1^2 + \delta q_2^2}$, and the relative phase difference, $y = \tan^{-1}\left(\frac{\delta q_1}{\delta q_2}\right)$. These two experimentally determined quantities, amplitude and phase, may be used to determine the two desired components of the differential wavevector, δq_1 and δq_2. Therefore, in our approach we have the full set of information needed to quantify pump-induced changes to the complex wavevector from raw data of ΔS, bypassing a Fourier Transform analysis. Importantly measurements of S_r and ΔS are carried out simultaneously[2] minimizing the error in our procedure.

Supplementary Note 2: "Angular corrections of the wavevector"

Supplementary Figure 1 shows the propagating waveguide mode that is detected by near-field imaging. Fringes are observed with a wavelength of λ_{obs} due to the interference of beams with the electric fields $\mathbf{E}_1$ and $\mathbf{E}_2$, produced by beam paths #1 and #2 in Supplementary Fig. 1b (see Supplementary Note 1). The in-coming laser beam is focused to the apex of the tip with incident angles α and θ with respect to horizontal plane and sample edge respectively. Laser beam #1 is back-scattered directly by the tip and registered on the detector. The tip also launches a waveguide mode which travels to the sample edge and scattered out as beam #2. Momentum conservation along the sample edge requires that the traveling direction of the waveguide mode makes an angle β with respect to normal direction of sample edge, which satisfies the following momentum conservation equation:

$$\frac{2\pi}{\lambda_{\mathrm{p}}}\sin(\beta) = \frac{2\pi}{\lambda_0}\cos(\alpha)\cos(\theta) \qquad (8)$$

$$\beta = \sin^{-1}\left(\frac{\lambda_{\mathrm{p}}}{\lambda_0}\cos(\alpha)\cos(\theta)\right) \qquad (9)$$

where λ_{p} and λ_0 are the wavelengths of the waveguide mode and free-space light respectively. In our set-up the angle α is fixed at approximately $30°$. The two beams accumulate a relative phase difference, $\Delta\phi$, as the waveguide mode travels in WSe_2. The difference in the emission position of the scattered light satisfies:

$$\Delta\phi = \frac{2\pi}{\lambda_{\mathrm{p}}}\frac{x}{\cos(\beta)} - \frac{2\pi}{\lambda_0}\cos\left(\frac{\pi}{2} - \theta - \beta\right)\frac{x}{\cos(\beta)}\cos(\alpha) \qquad (10)$$

where x is the distance between the tip and sample edge. Thus, $\Delta\phi = \frac{2\pi}{\lambda_{\mathrm{obs}}}x$ and

$$\lambda_{\text{obs}} = \lambda_{\text{p}}\left(\frac{1}{\cos(\beta)} - \frac{\lambda_{\text{p}}}{\lambda_0}\frac{\cos(\alpha)\sin(\beta+\theta)}{\cos(\beta)}\right)^{-1} \quad (11)$$

is the wavelength observed in a near-field imaging experiment. In the perpendicular configuration $\theta = 90°$ and $\beta = 0$. The observed oscillation wavelength is given by, $\lambda_{\text{obs}} = \lambda_{\text{p}}\left(1 - \frac{\lambda_{\text{p}}}{\lambda_0}\cos(\alpha)\right)^{-1}$. Thus, our formulas reduce to a form that is in good agreement with previous reports[5]. We recognize that the observed wavevector $q_{\text{obs}} = 1/\lambda_{\text{obs}}$ must be corrected for angle of incidence using Supplementary Equation 11 to extract the angular corrected wavevector of the waveguide mode $q_{1,\text{l}} = 2\pi/\lambda_{\text{p}}$. The subscript l = r, or p indicates the reference and photo-excited wavevector respectively.

We now present data taken to experimentally test the model of angular correction described above. In Supplementary Fig. 2a we show the near-field scattering amplitude as a function of the real space co-ordinate, *x*, relative to the edge of the flake at *x* = 0. The measurement is repeated on the same flake for a series of different angles of incidence. The Fourier transforms of these data are shown in Supplementary Fig. 2b. A maximum at $\lambda_{\text{obs}}^{-1}$, is observed in the Fourier transform. The observed wavevectors of the TM_0 mode are marked with the black arrows. The observed wavelength is plotted against the angle, θ, in Supplementary Fig. 2c. The solid line is produced with Supplementary Equation 11 using λ_{p}=470 nm, which is the angular corrected wavelength of the waveguide mode. Good agreement with Supplementary Equation 11 is established with the data presented in Supplementary Fig. 2.

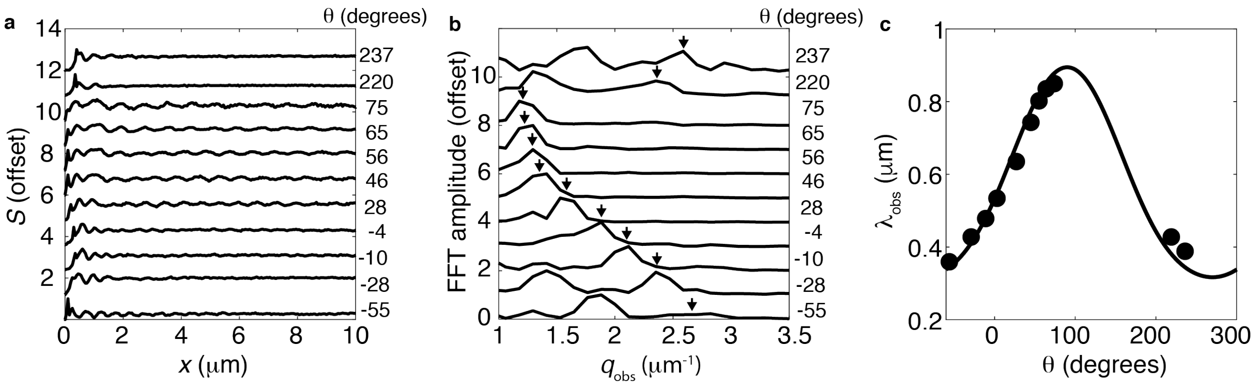

Supplementary Figure 2| Angle dependence of the polaritonic wavevector. a, Raw data taken on a 90 nm WS_2 flake on Al_2O_3 substrate with the probe energy of *E*=1.46 eV after rotating the sample at a series of angles, *θ*. **b**, Fourier transforms of the data in panel **a**. The arrows indicate the real component of the observed *q*-vector, q_{obs}. **c**, The observed wavelength of the waveguide mode extracted from the Fourier transform analysis is shown at a series of probe angles. The solid line is a fit using Supplementary Equation 11.

For the configuration used in the main text, $\theta \cong 55 +/- 5°$. In our modeling we applied Supplementary Equation 11 directly to extract the wavevector, $q_{1,\text{l}}=2\pi/\lambda_{\text{p}}$, from the raw data where the subscript l = r, or p indicates if the data were collected at equilibrium or under photo-excited conditions, respectively. In the main text the Fourier transforms of the raw data (Supplementary Fig. 2b) are displayed in angular corrected

units where we mapped the value of q_{obs} to $q_{1,l}$ at each point of frequency space using Supplementary Equation 11. The latter, angle-corrected wavevector, is displayed and compared with calculation throughout the main text and supporting information. The imaginary component of the wavevector, presented in Fig. 3a of the main text, was not corrected for angle of incidence.

Supplementary Note 3: "Relationship between q-vectors and the dielectric function"

In this Section we establish the quantitative relationship between the q-vector of the waveguide mode and the dielectric function of the sample/substrate system. To calculate the dispersion relationship of the waveguide mode we utilize Supplementary Equation 12, which was reported in Ref. [4]. Here we quote the result that TM modes satisfy the transcendental equation:

$$\sqrt{\frac{\varepsilon_{\mathrm{ab}}}{\varepsilon_{\mathrm{c}}}}\sqrt{\varepsilon_{\mathrm{c}}k_0^2-q^2}\,d=\tan^{-1}\left(\frac{\sqrt{q^2-\varepsilon_{\mathrm{sub}}k_0^2}\,\varepsilon_{\mathrm{ab}}}{\sqrt{\frac{\varepsilon_{\mathrm{ab}}}{\varepsilon_{\mathrm{c}}}}\sqrt{\varepsilon_{\mathrm{c}}k_0^2-q^2}\,\varepsilon_{\mathrm{sub}}}\right)+\tan^{-1}\left(\frac{\sqrt{q^2-k_0^2}\,\varepsilon_{\mathrm{ab}}}{\sqrt{\frac{\varepsilon_{\mathrm{ab}}}{\varepsilon_{\mathrm{c}}}}\sqrt{\varepsilon_{\mathrm{c}}k_0^2-q^2}}\right)+\mathrm{n}\pi \qquad (12)$$

where $\varepsilon_{\mathrm{ab}}$ is the complex dielectric function in the plane of the WSe_2 crystal, while ε_{c} is the complex dielectric function in the c-axis of WSe_2. The wavevector of the infrared radiation in free space is k_0, and the sample thickness is d. The sample is surrounded by air and a substrate with dielectric function $\varepsilon_{\mathrm{sub}}$.

In our experiments we consider the case of small light-driven perturbations altering the material properties. In the case that $\varepsilon_{\mathrm{ab}} \to \varepsilon_{\mathrm{ab}} + \Delta\varepsilon_{\mathrm{ab}}$, the wavevector will satisfy Supplementary Equation 12 with $q \to q + \delta q$. It is straightforward to show that:

$$\delta q \cong \kappa \cdot \Delta\varepsilon_{\mathrm{ab}} \quad (13)$$

in the limit $\Delta\varepsilon_{\mathrm{ab}} \ll \varepsilon$. Thus, small perturbations of the wavevector reveal an approximate linear relationship with the dielectric function. The coefficient, κ, depends on the probe energy, sample thickness, equilibrium dielectric constant of the sample and environment. We numerically calculate the value of κ with the aid of Supplementary Equation 12 to determine changes in the dielectric function of WSe_2 from our measurements of the complex wavevector. We emphasize that the latter description is valid if the effective optical constants of WSe_2 do not change during the course of waveguide propagation. If the optical constants do change while the waveguide mode propagates through the WSe_2 flake, we expect that the waveguide mode energy will be altered, which can cause changes of the phase velocity that are not captured by Supplementary Equation 13.

Supplementary Note 4: "Equilibrium spectroscopy"

We proceed to discuss data and calculations of the energy-momentum (E vs. q) dispersion relationship at equilibrium. The raw data of our experiment (Supplementary Fig. 3a) are Fourier transformed to produce traces in Supplementary Fig. 3b. The maxima of each peak in the Fourier transform (FT) correspond to the observed wavevector, $q_{obs} = \lambda_{obs}^{-1}$ (Section 3) of a waveguide mode (red and black dots). Two peaks are observed in FT traces. We extract the real part of the wavevector, $q_{1,r}$, from the peak positions of the FT while the imaginary component of the wavevector, $q_{2,r}$, is proportionate to the full width half maximum of the peak[5]. The experimentally determined wavevectors are corrected for the angle of incidence, as described in Supplementary Note 2, and plotted in Supplementary Fig. 3c. The low frequency mode (red dots) is in reasonable agreement with the trivial "air mode" with wavelength equal to that of radiation propagating in free space with $\lambda_p = \lambda_0$, consistent with previous reports[4]. We emphasize that the observed wavelength of the air mode is given by $\lambda_0 \left(\frac{1}{\cos(\beta_0)} - \frac{\cos(\alpha)\sin(\beta_0-\theta)}{\cos(\beta_0)}\right)^{-1}$ where $\beta_0 = \mathrm{asin}\,(\cos(\alpha)\cos(\theta))$ (see Supplementary Equation 11 section 3). Excellent agreement between the observed dispersion of the high frequency waveguide mode (black circles) and the calculated dispersion of the TM_0 waveguide mode that propagates in the WSe_2 crystal is evident in Supplementary Fig. 3c, as we discuss in detail in the next paragraph. The air mode is inevitably mixed with the TM_0 waveguide mode in the real-space fringe profiles. Nevertheless, the air mode is well separated from the TM_0 waveguide mode in the frequency domain, which allows us to separate these two modes. Furthermore, photoexcitation is only observed to impact the wavevector of the TM_0 waveguide mode. As such, we neglect the air mode in our analysis. After extracting the values of $q_r = q_{1,r} + iq_{2,r}$ from the FT analysis (Supplementary Fig. 3b) the wavevector of the TM_0 mode, q_r can be used to fit the real-space line profiles of nano-imaging data (Supplementary Fig. 3a). Utilizing Supplementary Equation 3 we produce the solid red lines in Supplementary Fig. 3a with the phase of the waveguide mode, φ, as the sole fit parameter. Thus, we are able fully characterize the q-vectors and phase of the TM_0 waveguide mode in equilibrium.

The imaginary part of the momentum-dependent reflectance co-efficient, $\mathrm{Im}\{R_p\}$, governs the dispersion relationship of the WSe_2 crystal and uncovers the parameter space for light propagation within the sample/substrate system. We display calculations of $\mathrm{Im}\{R_p\}$ in Supplementary Fig. 3c obtained using the Lorentz model for the dielectric function of WSe_2 (Section 8). Defining $2\pi k = q_1$ and using the known index of refraction of the SiO_2 substrate, $n_{SiO2}=1.46$ we highlight three features, which are apparent in the calculations shown in Supplementary Fig. 3: (1) A vertical line at k_0 (green line); (2) A vertical line at $k_0 \cdot n_{SiO2}$ (yellow); (3) A third maximum is observed at still higher momenta with a non-monotonic relationship between the dominant wavevector and probe energy. We overlay the analytic solution for the dispersion of the TM_0 waveguide mode, Supplementary Equation 12, on the $\mathrm{Im}\{R_p\}$ calculations (white line). The above trends in the dispersion relationship are identical between the two calculation methods. The feature (3) is, therefore, assigned to TM_0 waveguide modes that propagate through the slab of WSe_2, which are the focus of the main text.

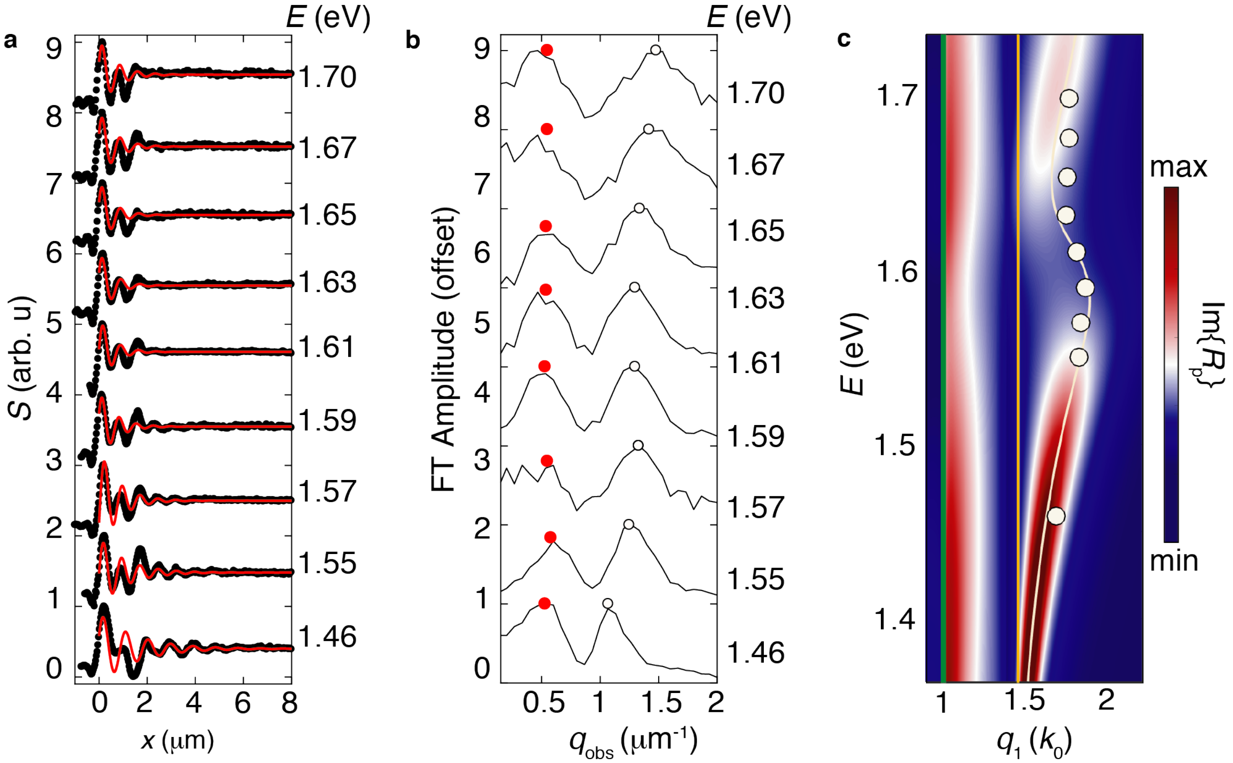

Supplementary Figure 3| Equilibrium dispersion relationship. **a**, Near-field amplitude at several probe energies plotted as a function of real-space position relative to the edge of the WSe_2/SiO_2 interface. The WSe_2 crystal is 90 nm thick. The solid red lines are fits to Supplementary Equation 3. **b**, Fourier transforms of the data shown in panel **a**. The curves displayed in panels **a** and **b** are vertically offset for clarity. The red dots indicate the dominant wavevector of the air mode. The black circles indicate the dominant wavevector, $q_{1,r}$, of the TM_0 waveguide mode, which propagates in WSe_2. **c**, The imaginary component of the p-polarized reflection coefficient, $\mathrm{Im}\{R_p\}$ is shown by the false color map as a function of frequency and momentum. The dispersion relationship calculated analytically using Supplementary Equation 12 is displayed with the white line. The calculations were produced with the Lorentz model for the dielectric function (Section 8). The green and yellow lines indicate the light cones of air and SiO_2 respectively as described above. The trend of $q_{1,r}$ vs. ω from the data (black dots with white interior) are overlaid.

The interaction of the waveguided probe radiation with excitons in the WSe_2 crystal is manifest in dispersion relationship of the waveguide mode (Supplementary Fig. 3). We emphasize that the dielectric function of bulk WSe_2 is highly anisotropic but remains positive throughout the investigated range of the electromagnetic spectrum[8] (1.45-1.8 eV). In the case that the real part of the permittivity is positive, the iso-frequency surface of the anisotropic crystal is a closed ellipse. Abbe's law of diffraction applies to such a system and the *q*-vectors of the waveguide mode consequentially reside within the material's light cone[9], which is in good agreement with our experimental results.

Supplementary Note 5: "Transient dynamics of the complex wavevector"

Raw data for the time-dependence measurements (Fig. 3 of the main text) are presented in this Section. The center frequency of the probe is red-detuned by approximately 5 meV from the center frequency of the A-exciton. In Supplementary Fig. 4 we report the dynamics of the near-field amplitude in the non-equilibrium case, $S_p(\Delta t)$, where Δt is the time-delay between the pump and probe pulses. In Supplementary Fig. 4a, we display

the pump-probe data collected at a time delay, Δt=-70 fs, along with a reference trace (Section 4) for comparison. The minus sign indicates that the peak of the pump intensity arrives at the sample 70 fs after the maximum of the probe intensity. We emphasize that the pump-probe convolution, approximately 160 fs, is much longer than 70 fs. At the time delay Δt=-70 fs the pump-intensity is, therefore, finite but not at its maximum value. The line profiles obtained for the photo excited and reference cases both reveal characteristic oscillations. An increase of the decay length with minor changes in the period, as compared with the equilibrium data, are observed at Δt=-70 fs. At a later time delay of Δt=330 fs, an enhancement of the decay length is no longer clearly observed. Pump-induced changes in the period of the oscillations are, however, evident. We chose to display data at these two representative time delays because the pump intensity is substantial at the former time delay (Δt=-70 fs) and negligible at the latter time delay (Δt=330 fs). The pump-induced changes are clearly revealed in the differential signal, ΔS, which is shown in Supplementary Figs. 4c, and d. Notably, a π/2 phase shift of the oscillations in ΔS is observed between the data collected at these two-time delays. These findings indicate a rapid decrease of δq_2 has occurred, which promptly recovers (see Supplementary Equations 6 and 7). In Supplementary Fig. 5 we show autocorrelation data used to determine the pump-probe convolution under conditions that were similar to those used in the experiment reported in the main text Figs. 3 a and b and in Supplementary Fig. 4. The dynamics of δq_2 are in agreement with the pump-probe convolution.

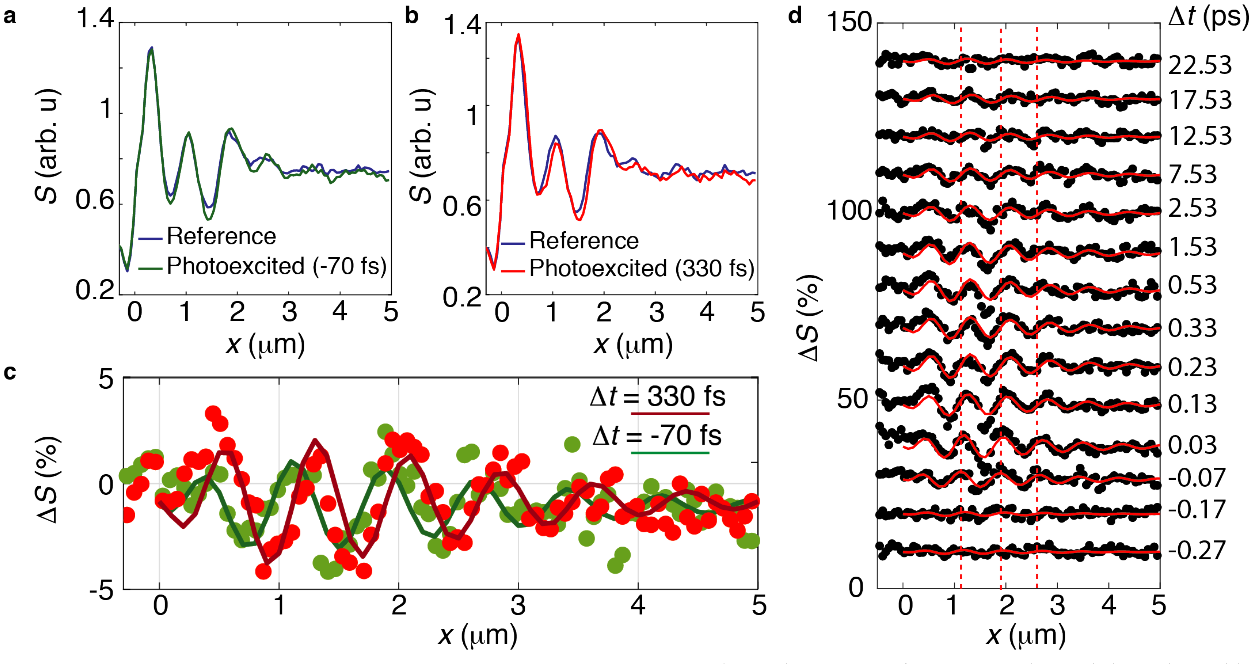

Supplementary Figure 4| Pump-probe dynamics of a WSe_2 crystal. The probe energy of E=1.61 eV is used throughout this study while a pump with energy of E=1.56 eV and power of 1.5 mW was applied to photo-excite a 90 nm thick WSe_2 crystal. **a** and **b**, Near-field amplitude as a function of real-space position relative to the edge of the WSe_2/SiO_2 interface. A reference trace of the near-field amplitude at equilibrium (blue) is shown. **a**, The near-field amplitude recorded with photo-excitation near pump probe overlap, while the intensity of the pump is non-zero (Δt = -70 fs), is shown in green. **b**, identical to panel **a**, except the photo-excited trace (red) is collected at Δt = 330 fs – a time-delay where the probe arrives after the pump intensity has completely decayed. **c**, Subtracting the reference data from the photo-excited data yields the differential ΔS signal, which is normalized to the mean value

of the reference data in the interior of the WSe_2 crystal. **d**, The differential signal, ΔS, recorded at a series of pump probe time delays are shown with the black dots. Fits constructed using Supplementary Equation 5 with the extracted values of δq_1 and δq_2 reported in Figs. 3**a** and 3**b** of the main text are overlaid with the solid red curves. The vertical red dashed lines indicate the locations where maxima are observed in line profiles of the waveguide mode at equilibrium, blue curves displayed in panels **a** and **b**. We emphasize that the $\gamma \cong \pi/2$ phase shift (see Supplementary Equation 7), clearly observed at Δt=330 fs in panels **c**, develops within three hundred femtoseconds of the photo-excitation event. **g** The curves displayed in panel **d** are vertically offset for clarity.

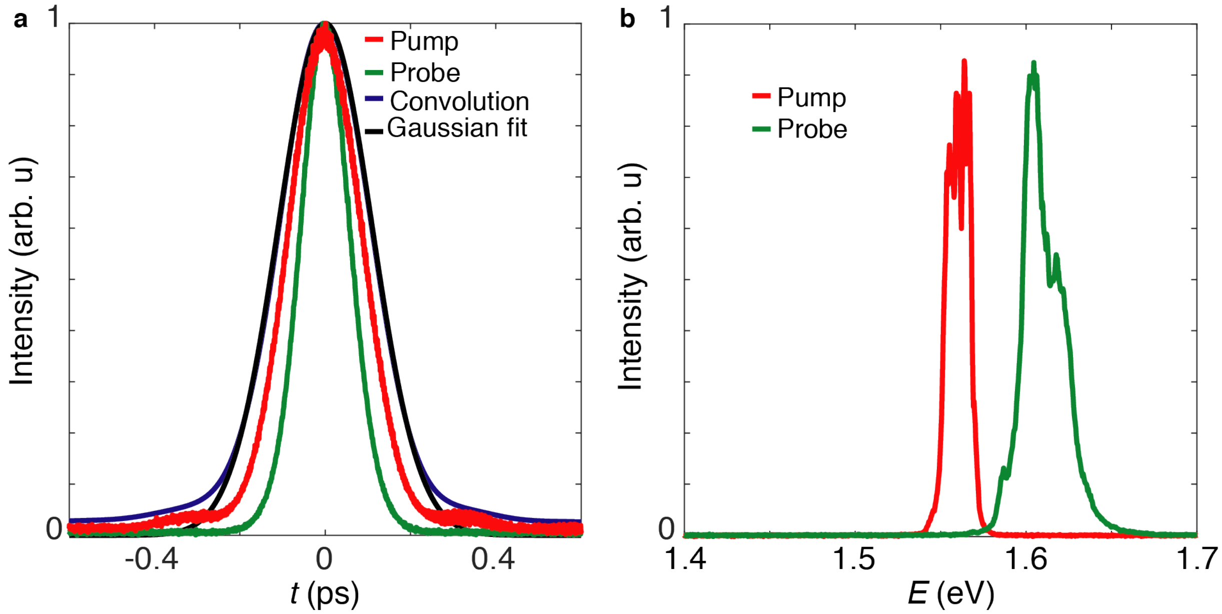

Supplementary Figure 5| Pulse characteristics for the pump probe experiment. a, autocorrelation measurements of the pump and probe pulses are shown by red and green curves respectively. The pump-probe convolution (blue) is fit to a gaussian function (black) to determine the pump-probe convolution, approximately 160 fs. **b**, Spectral intensity of the pump and probe pulses are shown with red and green curves respectively. The full width at half maximum of the spectral intensity is approximately 16 and 20 meV for the pump and probe respectively.

Supplementary Note 6: "Fluence dependence of the complex wavevector"

We proceed to characterize how the photo-induced changes of WSe_2 evolve with the pump power, P, and photon energy of our pumping laser. First, we note that P = 2 mW corresponds to a 5 nJ energy per pump pulse at our repetition rate of 377 kHz. Using the calculated area of the 9x16 μm^2 elliptical pump beam we estimate the maximum fluence applied to WSe_2, which is approximately 1 mJ cm^{-2}. The probe energy is fixed at approximately 1.61 eV, which is slightly red detuned from the A-exciton resonance (Supplementary Table 1). At pump-probe overlap (Δt=0) we study the pump-induced changes of the propagation length of the waveguide mode, δq_2. We find that δq_2 follows a linear trend against the pump power, P, with fixed pump-photon energy (solid lines). The measurement is repeated for two values of the detuning, Δ, defined as the energy of the pump relative to the energy of the A-exciton. The magnitude of δq_2 increases as Δ is decreased at constant P. These data collapse onto a single curve when the pump-induced change in wavevector is plotted against P/Δ (Supplementary Fig. 6). The experimentally measured scaling of the pump power and energy is, therefore, consistent with the trend anticipated for the optical stark effect[10].

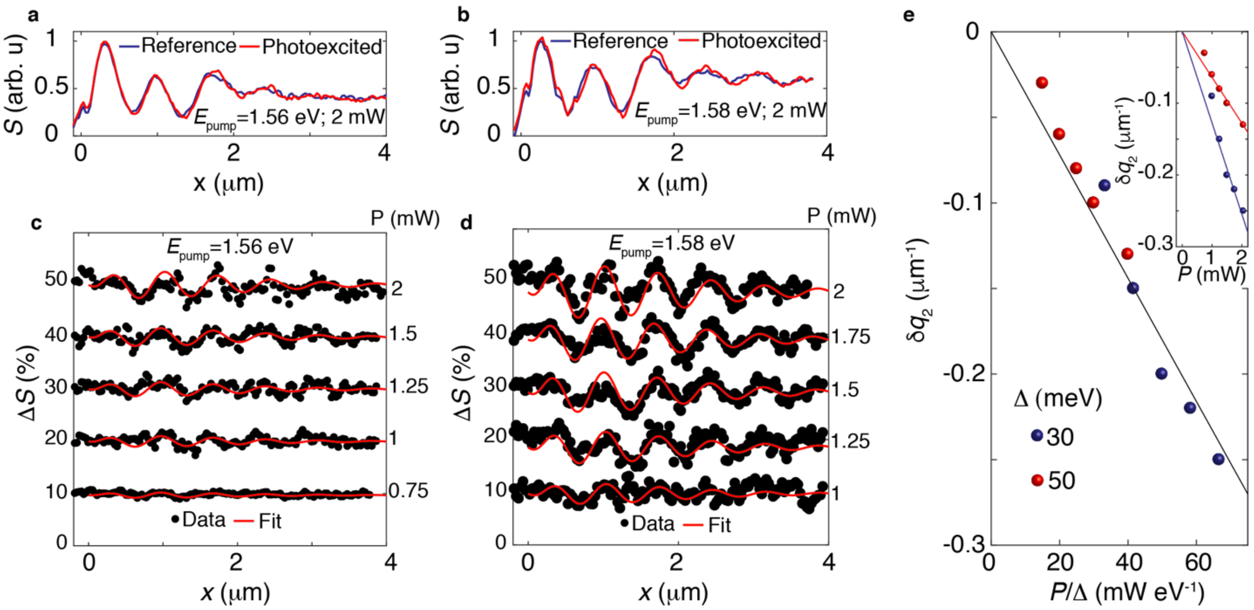

Supplementary Figure 6| Fluence dependence of the pump-probe response of WSe_2. The 90 nm thick WSe_2 crystal was investigated with a fixed probe photon energy of E = 1.61 eV at pump probe overlap (Δt = 0) for this study. The pump pulse has a bandwidth of approximately 15 meV. **a** and **b**, Line profiles of the near-field amplitude, S, under photoexcitation (red) are shown together with a reference trace (blue). **a**, Photo-excited data collected with a pump photon energy of E_{pump}=1.56 eV and pump power of P=2 mW and **b**, Photo-excited data collected with a pump photon energy of E_{pump}=1.58 eV and pump power of P=2 mW. **c** and **d**, Differential change in the near-field amplitude, ΔS, at a series of pump powers. The black dots are data, while the fits, constructed using Supplementary Equation 5 with the extracted values of δq_2 shown in panel **e**, are overlaid with red solid lines. **c**, Data were collected with the pump energy of E_{pump}=1.56 eV and **d**, Data collected with the pump energy of E_{pump}=1.58 eV. The curves shown in panels **c** and **d** are vertically offset for clarity. **e**, The detuning, $\Delta = E_A - E_{pump}$, is defined as the energy difference of the pump relative to the resonant energy of the A-exciton. The extracted values of δq_2 plotted against the rescaled x-axis, defined as power over detuning P/Δ. The extracted values of δq_2 are shown against the applied pump power in the inset.

Supplementary Note 7: “Pump-probe spectroscopy and transient nano-imaging”

The raw ΔS data for the pump probe spectroscopy measurements are displayed with black dots in Supplementary Fig. 7. Throughout our experiment the pump conditions were fixed (pump photon energy E=1.56 eV and pump power of 3 mW) while we the probe energy was varied. We highlight several significant features that are observed in the raw data. First, characteristic oscillations in line-profiles of ΔS are observed at all probe frequencies. The amplitude of these oscillations, A, is proportionate to the differential change of the wavevector (Supplementary Note 1). Second, as the probe energy is increased from 1.46 eV to 1.57 eV substantial increases of the amplitude A are observed. The rapid increase of A with increasing probe energy is followed by a non-monotonic decrease of A when the probe energy is slightly red-detuned from the A-exciton resonance. Finally, differences between the differential data collected at the two-time delays, Δt=1 ps and Δt=0, are also most apparent when the probe photon energy is nearly resonant with the A-exciton. The differential change in periodicity, δq_1, reported in Fig 3e of the main text were extracted from the line-profiles of ΔS using Supplementary Equation 5. Our study reveals a clear dispersion anomaly in the vicinity of the A-exciton with significant sub-ps dynamics exposing photo-induced transformations of the A-exciton.

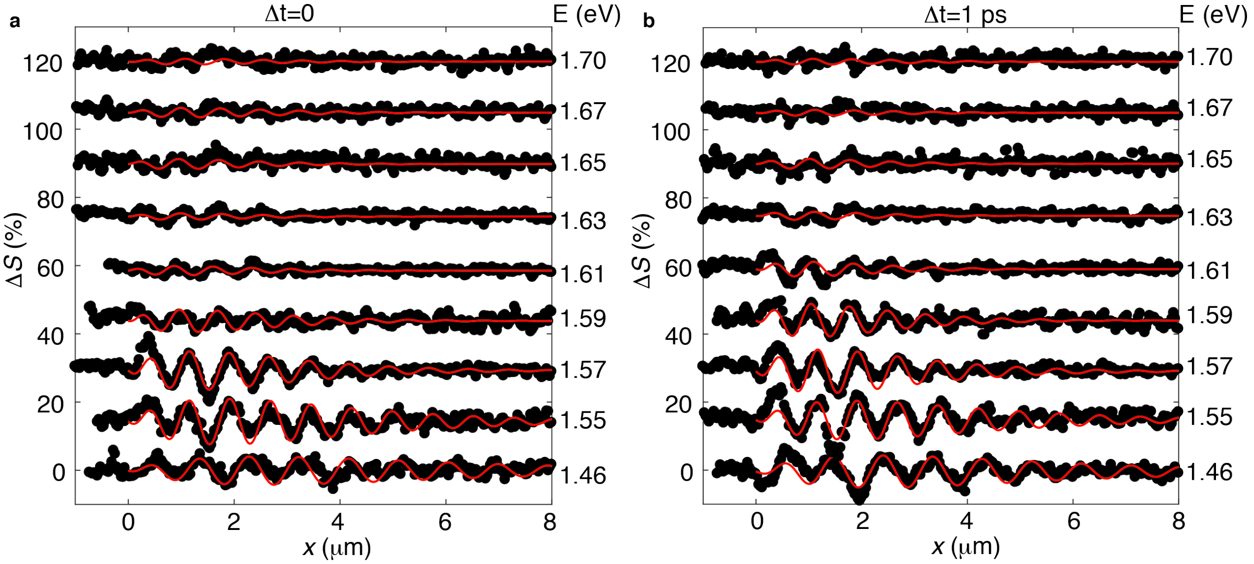

Supplementary Figure 7| Non-equilibrium spectroscopy and nano-imaging. These data were collected on a 90 nm slab of WSe_2 photo-excited with a pump energy E=1.56 eV and pump power of 3 mW **a**, Line profiles of the differential change in near-field amplitude ΔS at a series of probe energies collected at pump-probe overlap (Δt=0). The black dots are data while the fits constructed using Supplementary Equation 5 with the extracted values of δq_1 and δq_2 are overlaid in red. **b**, Identical to panel **a**, except with a pump probe time delay of Δt = 1 ps. The curves in panels **a** and **b** are vertically offset for clarity.

Supplementary Note 8: "Modeling the equilibrium dielectric function"

In this Section we discuss our model for the dielectric function in equilibrium. The salient features of the dielectric function are accurately re-produced with a model based on a series of Lorentzian oscillators:

$$\varepsilon_{ab} \cong \varepsilon_{\mathrm{stat}} + \sum_{\mathrm{j}=1}^{\mathrm{N}} \frac{f_{\mathrm{j}}}{(E_{\mathrm{j}})^2 - E^2 - \mathrm{i}E\gamma_{\mathrm{j}}} \qquad (14)$$

The parameters of the six oscillators considered in the Lorentz model are reported in Supplementary Table 1. The parameters $\varepsilon_{\mathrm{stat}}$=15.5 and the out of plane dielectric function ε_{c}=8 are also used. Our measurements were conducted over a limited range of probe energies (1.45 eV < E < 1.7 eV). Thus, only the A-exciton is directly observed in the experimental energy window. In the investigated range of energies, a further approximation is valid:

$$\varepsilon_{ab} \cong \varepsilon^{*}_{\mathrm{stat}} + \frac{f_1}{(E_1)^2 - E^2 - \mathrm{i}E\gamma_1} (15)$$

In Supplementary Equation 15 the offset of the real part of the dielectric function, $\varepsilon^{*}_{\mathrm{stat}}$ includes contributions from the static dielectric function and from excitons at high energies outside the investigated range of probe energies. In Supplementary Fig. 8 we display the dielectric computed with Supplementary Equations 14 and 15. All significant spectral features are accurately represented in the energy scale of 1.45 - 1.8 eV where our investigation of the waveguide mode was performed. In the main text,

Supplementary Equation 14 was used in our model calculations of the dispersion relationship in equilibrium (Fig. 2d of the main text), we remark that the results using Supplementary Equation 15 are nearly identical.

The simpler single oscillator model Supplementary Equation 15 was used in our non-equilibrium analysis (Fig. 3 of the main text). In the single oscillator model photo-induced changes to high-energy excitons that are outside the range of energies we investigated, and are not directly measured, are accounted for with the dispersion-less offset of the dielectric function, $\varepsilon^{*}_{\mathrm{stat}}$. Thus, we report only the specific perturbations to the A-exciton, which are directly extracted from our measurements in Table 1 of the main text. We remark that apart from a slight dispersionless offset, the in-plane component of the dielectric function constructed from the Lorentz model, (Supplementary Table 1; and Supplementary Equation 14), is in excellent agreement with the data of Li et al., reported in Supplementary Ref. [8].

Supplementary Table 1: Parameters for the equilibrium dielectric function of WSe_2:

	j=1	j=2	j=3	j=4	j=5	j=6
E_j (eV)	1.612	1.8	2.18	2.6	2.8	3
γ_j (meV)	106.8	251.9	258.4	248	1,264.8	1,248
f_j (eV^2)	1.2046	0.5337	2.081	1.0608	15.4973	20.9515

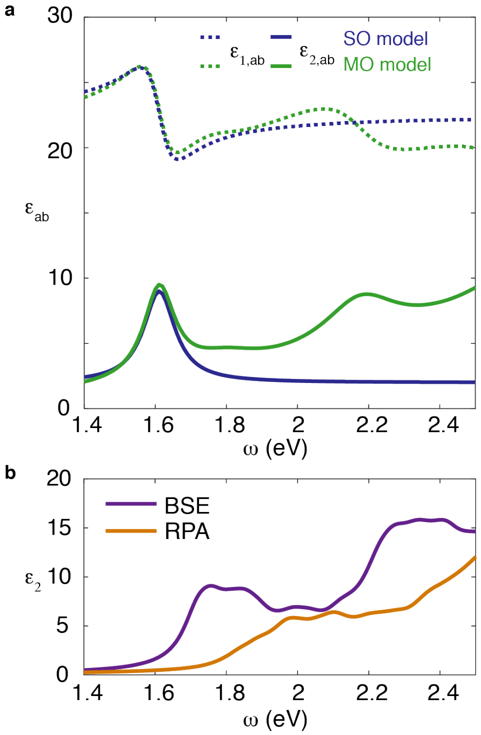

Supplementary Figure 8| The equilibrium dielectric function of WSe_2. **a**, All dashed curves display the real part of the permittivity, $\varepsilon_{1,\mathrm{ab}}$, while all solid curves display the imaginary part of the permittivity, $\varepsilon_{2,\mathrm{ab}}$. Fits using a multi-oscillator (MO) Lorentz model (Supplementary Equation 14; Supplementary Table 1), representing multiple excitonic transitions are shown with green curves. Fits with a single oscillator (SO) Lorentz model representing the A-exciton (Supplementary Equation 15; Table 1 at equilibrium) are shown with blue curves. **b**, First principles calculations of the dielectric function. The values for $\varepsilon_{2,\mathrm{ab}}$ calculated with the Bethe-Salpeter equation (BSE) are shown in purple while the values for $\varepsilon_{2,\mathrm{ab}}$ calculated within the Random Phase Approximation (RPA) are shown with the orange curve.

The dielectric response of WSe_2 is also obtained from first-principles calculations based on the GPAW code[11, 12] (Supplementary Fig. 8b). The imaginary part of the dielectric function is obtained from Bethe-Salpeter Equation (BSE) using 2 valence and 2 conduction bands based of the LDA functional, 150 eV cut-off energy for the static screened interaction and a 45x45x3 k-point grid. The data displayed in the main text were offset by the A-exciton energy, defined as the energy where the first maxima of ε_2 is observed. The BSE results contain spectral weight from excitons in addition to spectral weight from band-to-band transitions. To estimate the spectral weight associated with excitons we also calculated the dielectric function with the random phase approximation (RPA), which excludes excitonic effects. To obtain the excitonic portion of the dielectric response we first calculate the variation of the imaginary part of the permittivity due to the excitonic effects and then map it to the real part of the permittivity through the Kramers-Kronig relations in formulas:

$$\varepsilon_{1,\mathrm{ab}}^{\mathrm{corr}}(\omega) = \varepsilon_{1,\mathrm{ab}}^{\mathrm{RPA}}(\omega) + \frac{2}{\pi}\int_0^{\omega_c} \mathrm{d}\omega' \, \frac{\delta\varepsilon_{2,\mathrm{ab}}(\omega)}{\omega'^2-\omega^2} \qquad (16)$$

Where $\delta\varepsilon_{2,\mathrm{ab}}(\omega) = \varepsilon_{2,\mathrm{ab}}^{\mathrm{BSE}}(\omega) - \varepsilon_{2,\mathrm{ab}}^{\mathrm{RPA}}(\omega)$ was defined. The integration was truncated at the frequency $\omega_c = 2.5$ eV. The results were insensitive to our exact choice of ω_c indicating

the calculation has converged. In terms of the index of refraction, $\varepsilon_{\rm BSE}(\omega) = \varepsilon_{\rm 1,ab}^{\rm RPA}(\omega) + \varepsilon_{\rm 1,ab}^{\rm corr}(\omega) + {\rm i}\varepsilon_{\rm 2,ab}^{\rm BSE}(\omega)$ includes excitonic contributions, while $\varepsilon_{\rm RPA}(\omega) = \varepsilon_{\rm 1,ab}^{\rm RPA}(\omega) + {\rm i}\varepsilon_{\rm 2,ab}^{\rm RPA}(\omega)$ does not. Finally, we are able to estimate the contribution to the index of refraction from excitons as $\varepsilon_{\rm EX}(\omega) = \varepsilon_{\rm BSE}(\omega) - \varepsilon_{\rm RPA}(\omega)$.

We emphasize that our first principles estimates are capable of separating spectral weight associated with band-to-band transitions from the spectral weight associated with excitonic transitions. The latter information, reported in Supplementary Fig. 10b, augment our experimental optical data, which are affected by both excitonic and interband effects. Our approach, therefore, allows us to accurately determine the maximum possible changes of the dielectric response that can be attained by targeted manipulation of excitons – a fundamental limit to the modulation of the optical properties within bulk WSe_2 crystals.

Supplementary Note 9: "Incoherent photo-induced transformations"

To investigate plausible roles of optically generated carriers in optical modulation we turn our attention to the mid-infrared spectral range. We observed the appearance of a "Drude-like" edge in WSe_2 at the pump-probe time delay Δt=2 ps (Supplementary Fig. 9). We use the Drude-Lorrentz model:

$$\varepsilon = \varepsilon_{\rm stat} + \Delta\varepsilon \cong \varepsilon_{\rm stat} - \frac{\omega_{\rm p}^2}{\omega^2 - \omega_0^2 - {\rm i}\omega\Gamma} \quad (17)$$

To model the dielectric function. In Supplementary Equation 17 the Drude limit is recovered by taking the resonant energy $\omega_0 \rightarrow 0$. We emphasize that Supplementary Equation 17 agrees with our data provided that $\omega_0 < 45$ meV. Thus, our measurement cannot distinguish between bound excitons and a pure Drude response from itinerant carriers. To simplify the model, we consider the Drude limit by setting $\omega_0 = 0$. To quantitatively extract $\omega_{\rm p}$ and Γ we employ the lightning rod model[13, 14, 15] which allows us to calculate the near-field amplitude (solid black curve in Supplementary Fig. 9). For simplicity the dielectric response was taken to be isotropic in our lighting rod model calculations. Under nominally similar photoexcitation conditions (pump laser with 820 nm center wavelength, 80 meV bandwidth, 2 mW excitation power, 750 kHz repetition rate) we extract $\omega_{\rm p}$ = 2,500 cm^{-1}. The scattering rate $\Gamma = 350\ {\rm cm}^{-1}$ is also extracted. As argued in the main text the decrease of the $\varepsilon_{\rm stat}$, observed in our waveguide experiments implies that a fraction of the optically generated carriers are itinerant. Under the extreme assumption that all optically generated carriers are itinerant we can extract the carrier density as[16]:

$$n = (0.724 \text{ x } 10^{21}\ {\rm cm}^{-3}) \left(\frac{m^*}{m_{\rm e}}\right) \left(\frac{\hbar\omega_{\rm p}}{8066}\right)^2 \qquad (18)$$

The extracted three dimensional free carrier density is n=$n_{\rm 3D}$=1.4x10^{19} cm^{-3}; in this analysis we assumed the effective mass of the direct band m^*=0.17 $m_{\rm e}$ in accord with

Ref. [17]. In terms of the carrier density per atomic layer n_{2D}, we obtained $n_{2D}=n_{3D}d_{WSe2}=1x10^{12}$ cm^{-2} assuming the atomic thickness of WSe_2 layers d_{WSe2}=0.7 nm (Ref. [18]). At comparable densities of itinerant carriers drastic changes in the optical spectra of monolayer and bilayer TMD systems have been reported[19, 20, 21, 22]. These transformations were attributed to the onset of a Mott transition where the electron-hole plasma screens the Coulomb interaction that binds excitons together causing the excitons to dissociate[23]. Estimates for the critical Mott density have been reported in a wide range from $3x10^{12}$ cm^{-2} [Ref. 21] to $1x10^{14}$ cm^{-2} [Ref. 19] in mono and bilayer TMD systems. Bulk three dimensional models predict a lower Mott-threshold, with a lower bound of approximately $3x10^{18}$ cm^{-3} [Ref. 24]. In our experimental data we observe about a 10% bleaching of the A-exciton, which is consistent with other experimental spectra taken near, but not significantly above, the Mott threshold[19, 20, 21]. Finally, we remark that as one approaches the Mott-transition the binding energy of excitons decreases. An interesting follow up study, suggested by our results, would be to investigate how the optical Stark effect is impacted as the binding energy of the A-exciton approaches zero in TMDs. The suggested study could be carried out by monitoring the optical Stark shift as the carrier density is tuned to, and beyond, the Mott threshold with photo-excitation[19, 21] or electrostatic gating[20].

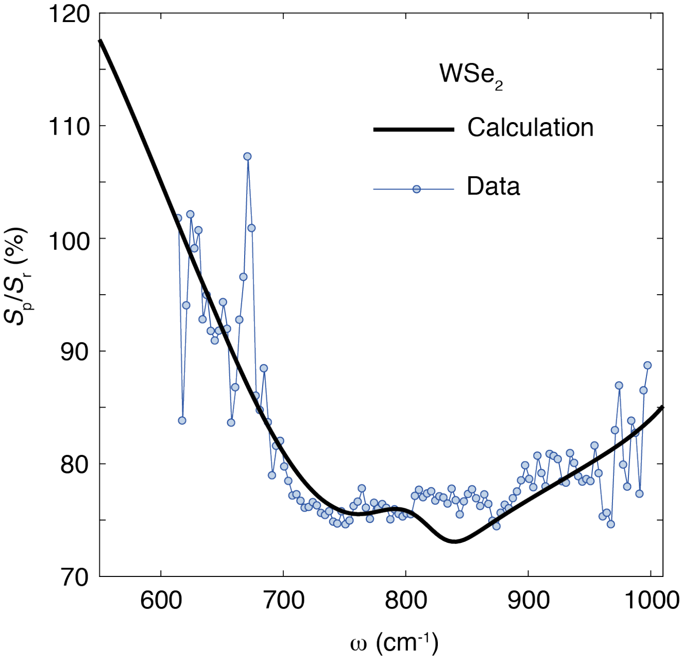

Supplementary Figure 9| Mid-IR spectroscopy. Near-field amplitude spectra recorded with the pump on, S_p, were normalized to spectra obtained with the pump off, S_r. The Lightning Rod Model[13] was used to produce the solid line as described in the text.

Following an abrupt increase of the periodicity of the waveguide mode, which is described by δq_1, the decay process is well described by Auger equation:

$$\frac{\mathrm{d}(\delta q_1)}{\mathrm{d}t} = -\eta \delta q_1^2 + A\exp[-t/\tau] \qquad (19)$$

Our motivation for using Supplementary Equation 19 is purely phenomenological. The fit presented as the blue line in Fig 3b of the main text yields η=1.2x10^{13} k_0^{-1} s^{-1}. The intensity, modeled with the pump-probe convolution, τ, (Supplementary Fig. 5) and an arbitrary amplitude, *A*, dictates the anticipated rise time (green curve in Fig. 3a of the main text). The constant pre-factor η can be regarded the decay rate of an annihilation processes, which may include decay by collision with defect or trap states and exciton-exciton annihilation[25, 26].

Supplementary Note 10: "Excitonic optical modulation and tunable birefringence within WSe2 waveguides"

Our transient images allow us to assess fundamental limits to excitonic vdW optical modulators. Strong modulation of the index of refraction near excitonic resonances has been discussed in several previous reports on TMD monolayers[27] and the potential for excitons to enhance optical modulation has been suggested[28]. Here, our transient nano-imaging experiment provides direct evidence that excitons do enhance optical modulation within a vdW semiconducting waveguide. We observed considerable changes of the phase velocity, the magnitude of $\Delta\varepsilon_{1,ab}$(E=1.45 eV) reached 1.3 in our experiments, which exceeds 5% of the value of the permittivity at equilibrium (Main text; Fig. 1). The data of the main text Fig. 3 may be recast as the spectra of differential changes of the in-plane component of the dielectric tensor, $\Delta\varepsilon_{1,ab}(E)$, which are displayed in Supplementary Fig. 10. To assess the potential to exploit excitons in optical modulation applications we computed the excitonic contribution to the index of refraction, ε_{EX}, from first-principles density functional calculations (see Supplementary Section 8). Our first principles results provide an upper bound of refractive changes that would be attained provided all excitons in WSe_2 were to dissociate reaches $-\Delta\varepsilon_{1,x} = \varepsilon_{EX} =$ 4.5, which exceeds 16% of the value of the ab-plane permittivity at equilibrium (Supplementary Fig. 10b).

Apart from $|\Delta\varepsilon_{1,ab}|$, losses within the waveguide are of paramount importance for devices. Remarkably, additional losses are unobservable in our pump-probe data even under the strongest photo-excitation conditions. In fact, losses can be suppressed by photoexcitation as witnessed by the negative sign of δq_2 (see main text Fig. 3a), albeit over a restricted frequency region in the vicinity of the A-exciton (Supplementary Fig. 10c). Notably, the magnitude of $\Delta\varepsilon_x$ is maximized near excitonic transitions, in the range of several eV, where added losses from itinerant carriers are exceedingly small in WSe_2. These photo-induced changes establish unique virtues of optically-induced modulation in vdW semiconductors that are rooted in manipulation of excitons[29, 30]. Provided that equilibrium loss governed by the excitonic linewidth is further reduced in WSe_2 and other vdW semiconductors, these systems could offer practical modulation solutions. Such a reduction of equilibrium loss may be possible given observations of ultra-narrow exciton linewidths in high purity transition-metal-dichalcogenides[31] at liquid nitrogen temperatures.

Additionally, we emphasize the significance of anisotropy in the modulation of the real component of the dielectric tensor, studied in this work. In Ref. [5] D. Hu et. al., have

noted that the strong anisotropy inherent to vdW semiconductors can be used to tune the modal birefringence in MoS_2 waveguides of varying thickness. Here, we note that the optical polarizability associated with excitons is highly anisotropic. Manipulation of excitons may, therefore, present an opportunity to control the optical birefringence of WSe_2 and other related vdW semiconductors on-demand. Finally, we stress that an encouraging degree of modulation is attained at fs time scales meeting the most stringent demands of ultra-fast optical circuits.

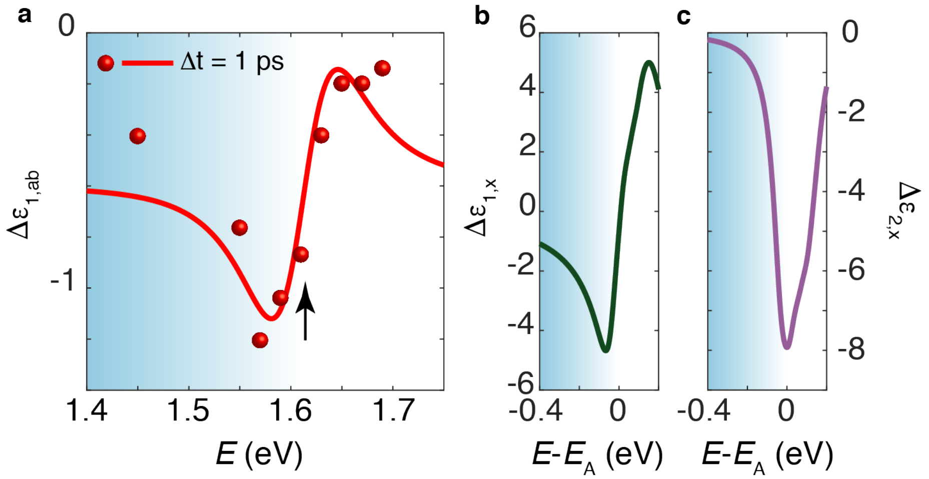

Supplementary Figure 10| Limits to excitonic optical modulators based on WSe_2. **a**, Experimental change in the permittivity within a WSe_2 waveguide, $\Delta\varepsilon_{1,ab}$ under fixed pumping conditions (dots) and Lorentz model calculations with identical parameters to those displayed in Fig. 3e of the main text (solid lines; see Table 1 of the main text). Data and Lorentz model calculations at the time delay of Δt = 1 ps are shown in red. **b**, The upper limit of changes of the real and **c**, imaginary components of the permittivity attainable from perturbing excitons in WSe_2 waveguides at room temperature obtained from first principles theory (green and purple curves respectively; see Supplementary Section 8). The transparency window of WSe_2 is indicated by the blue shaded region.

Supplementary Note 11: "Thickness dependence"

By measuring the complex wavevector as a function of the film thickness both the in-plane, $\varepsilon_{1,ab}$ and out of plane, $\varepsilon_{1,c}$, components of the dielectric tensor can be determined as shown in Ref. 4. The thickness dependence data in Supplementary Fig. 11, acquired with the probe energy of 1.45 eV, were used to extract the real component of the dielectric tensor:

$$\left|\left|\mathrm{Real}\{\varepsilon(E = 1.45\ \mathrm{eV})\}\right|\right| = \begin{bmatrix} 24.5 & 0 & 0 \\ 0 & 24.5 & 0 \\ 0 & 0 & 8 \end{bmatrix} \qquad (20)$$

We stress that the optical polarizability of $\varepsilon_{1,c}$ exhibits negligible dispersion within the investigated range of probe energies ($1.4 < E < 1.8$ eV)[32, 33].

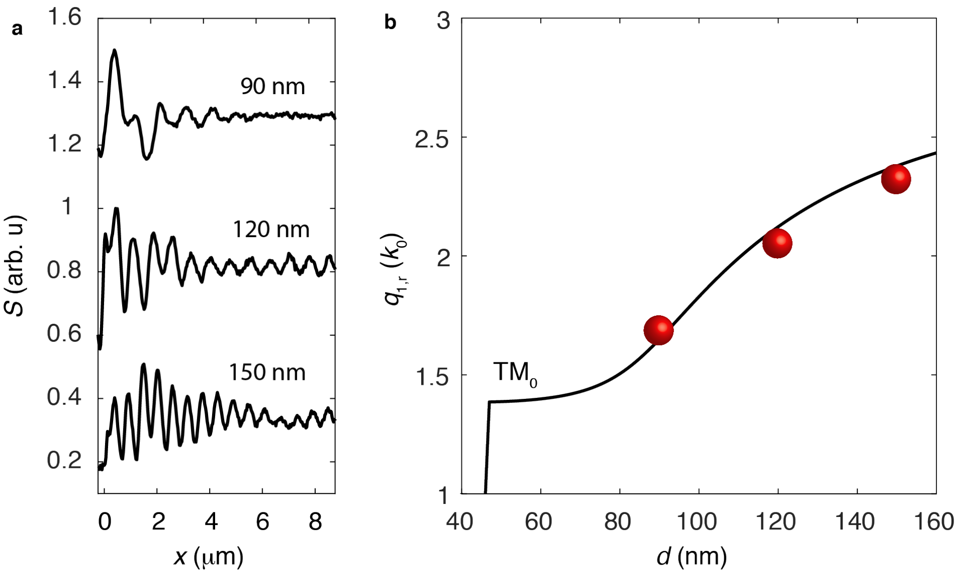

Supplementary Figure 11| Thickness dependence of the TM_0 waveguide mode. **a**, Experimental line profiles of S as a function of the real space position of the AFM probe, *x*, for flakes of three different thicknesses, *d*. **b**, The real component of the complex wavevector extracted from the data in panel **a** (red dots) are overlaid on a calculation using Supplementary Equation 12 for the TM_0 branch of the waveguide mode. The in-plane component of the dielectric function (given in Supplementary Section 8) at the probe energy of $E = 1.45$ eV as well as the out-of-plane component of the dielectric function $\varepsilon_c = 8$ are used in the calculation.

Supplementary Note 12: "Transient spectroscopy and imaging with broad-band pulsed lasers"

In our experiments, we employ broadband pulsed lasers. To account for the finite bandwidth of the laser pulse we consider a wave-packet:

$$E(\mathbf{r},t) = \int \mathrm{d}k\, M(k) \exp\left[\mathrm{i}(kx - \omega t)\right] \qquad (21)$$

In free space, *M*(*k*) is simply the momenta of the incident radiation. When the sharp tip of the AFM probe is illuminated by an infrared beam described by Supplementary Equation 21 the wave packet will be modified:

$$E_\mathrm{p}(\mathbf{r},t) = \int \mathrm{d}k\, B(k) \exp\left[\mathrm{i}(kx - \omega t)\right] \qquad (22)$$

The sharp apex of the AFM probe confines radiation to a length scale, a, equal to the radius of the probe (~20 nm). In turn the momentum response function of the AFM probe, *R*(*k*), facilitates access to momenta outside of the light-cone, a well-known feature that is described in previous works[13]. The properties of the sample, together with the response of the AFM probe, determine the distribution of momenta in the tip/sample system, *B*(*k*). In the present case, our WSe_2 samples support propagating TM_0 modes with momenta that are outside of the free-space light cone but inside the light-cone of WSe_2 (see Section 4). The broadband wave-packet launches multiple TM_0 waveguide modes simultaneously, which can interfere. Inserting a gaussian wave-packet $B(k) = v_\mathrm{g} \exp\left[-\frac{(k-k_0)^2}{(\Delta k / v_\mathrm{g})^2}\right]$ into Supplementary Equation 22 we recognize general, qualitative,

features that are expected when broadband light sources are employed. In the latter expression, k_0, is the momenta at the center of the wave-packet, Δk, characterizes the distribution of momenta within the wave-packet and, v_g, is the group velocity of the propagating mode. The length scale where reduced propagation length should be observed from these interference effects is given by $v_g/\Delta k$.

By varying the bandwidth of our probe channel, we experimentally investigate the theory described above. In Supplementary Fig. 12 we display line-profiles of the near-field amplitude as a function of the distance to the edge of the WSe_2 flake. A bandpass filter with full width at half maximum of about 15 meV (= 4 THz) was inserted to the beamline to narrow the bandwidth of the probe beam before it was delivered to the AFM microscope. With the latter, narrowband, illumination we recorded the red curve. When the bandpass filter was removed and the full bandwidth of the probe channel, 80 meV (= 20 THz) was employed we measured the line profile displayed with the black curve in Supplementary Fig. 12. It is apparent that the observed propagation length of the waveguide mode is dependent on the bandwidth of the probe beam. The quantity $v_g/\Delta \underline{k}$ defines the length scale where destructive interference within the wave-packet dominates the propagation length. In the former case of narrowband, 15 meV, illumination $v_g/\Delta k$=25 μm and the bandwidth of the wavepacket has a negligible impact on the experimentally measured line-profile within the 12 μm field of view we investigated. In the latter case of the broadband, 80 meV, illumination the anticipated propagation length is $v_g/\Delta k$=5 μm, which is in agreement with our observations.

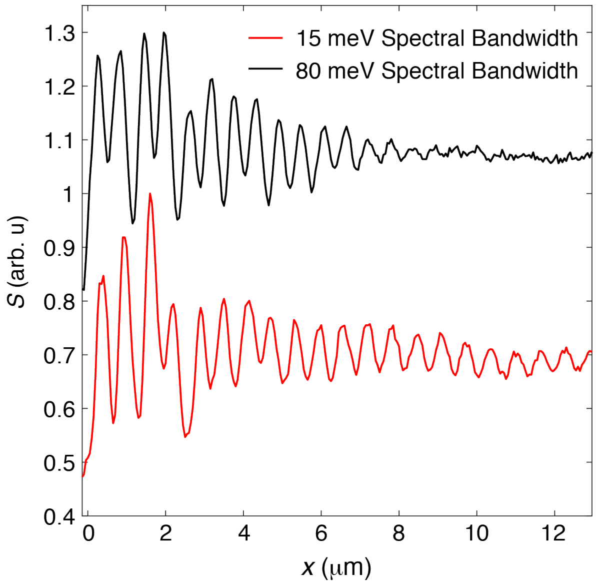

Supplementary Figure 12| Line profiles of the near-field amplitude obtained on a 210 nm thick WS_2 crystal on an SiO_2 substrate. A probe channel with energy of E=1.46 eV was used in this study. The black curve was recorded with the full spectral bandwidth of the laser, approximately 80 meV. The red curve was recorded with a spectrally filtered 15 meV bandwidth probe beam. The curves were vertically offset for clarity.

Supplementary Note 13: "Pump-probe dynamics in a WS_2 waveguide"

We observed substantial photo-induced optical modulation in a closely related dichalcogenide material WS_2 (Supplementary Fig. 13); the gross features of these latter pump-probe data are similar to our observations for WSe_2. At a fixed probe energy E=1.46 eV we recorded line-profiles of the near-field amplitude for a 210 nm thick WS_2 flake on the SiO_2 substrate. The oscillations that are observed in the line-profiles are dominated by the TM_0 waveguide mode, which propagates within WS_2. We repeated the measurement with photoexcitation at a fixed pump energy, E_p=1.88 eV at a series of pump powers (Supplementary Fig. 13a). We observed that the periodicity of the waveguide mode was significantly increased on photoexcitation. Pump induced changes of the decay length are, however, insignificant and remain below our detection limits at all pump powers. The pump-induced change in the periodicity, described by δq_1, increases linearly with the applied pump power with no apparent sign of saturation (Supplementary Fig. 13b).

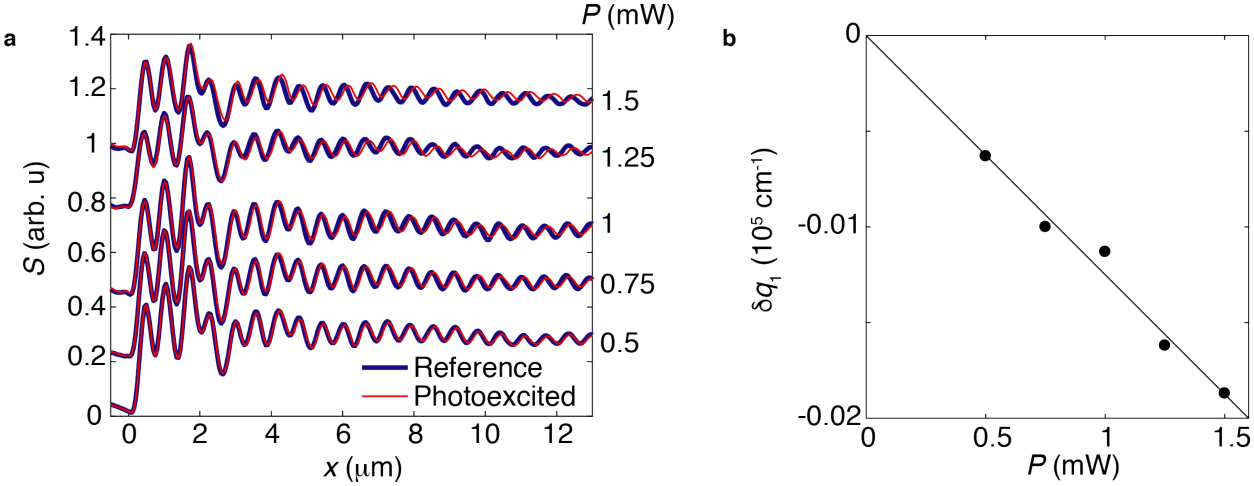

Supplementary Figure 13| Power dependence of the photo-excitation response for WS_2. A WS_2 crystal with thickness of 210 nm was investigated with a 1.46 eV probe pulse under photo-excitation with a 1.88 eV pump pulse at the time delay Δt = 0.1 ps in this study. **a**, Line-profiles of the near-field amplitude under photoexcitation (red) are shown together with a reference (blue) at a series of pump powers. The curves are vertically offset for clarity. **b**, The pump-induced change in the periodicity of the waveguide mode as a function of the pump power.

Supplementary References

1. Fei Z*, et al.* Gate-tuning of graphene plasmons revealed by infrared nano-imaging. *Nature* **487**, 82-85 (2012).

2. Sternbach AJ*, et al.* Artifact free time resolved near-field spectroscopy. *Opt Express* **25**, 28589-28611 (2017).

3. Hu D, Chen K, Chen X, Guo X, Liu M, Dai Q. Tunable Modal Birefringence in a Low-Loss Van Der Waals Waveguide. *Advanced Materials* **31**, 1807788 (2019).

4. Hu D*, et al.* Probing optical anisotropy of nanometer-thin van der waals microcrystals by near-field imaging. *Nature Communications* **8**, 1471 (2017).

5. Hu F*, et al.* Imaging exciton–polariton transport in MoSe2 waveguides. *Nature Photonics* **11**, 356 (2017).

6. Dai S*, et al.* Efficiency of Launching Highly Confined Polaritons by Infrared Light Incident on a Hyperbolic Material. *Nano Letters* **17**, 5285-5290 (2017).

7. Eisele M*, et al.* Ultrafast multi-terahertz nano-spectroscopy with sub-cycle temporal resolution. *Nature Photonics* **8**, 841 (2014).

8. Li Y*, et al.* Measurement of the optical dielectric function of monolayer transition-metal dichalcogenides: MoS2, MoSe2, WS2, and WSe2. *Physical Review B* **90**, 205422 (2014).

9. Narimanov EE, Kildishev AV. Naturally hyperbolic. *Nature Photonics* **9**, 214 (2015).

10. Sie EJ, McIver JW, Lee Y-H, Fu L, Kong J, Gedik N. Valley-selective optical Stark effect in monolayer WS2. *Nature Materials* **14**, 290 (2014).

11. Enkovaara J*, et al.* Electronic structure calculations with GPAW: a real-space implementation of the projector augmented-wave method. *Journal of Physics: Condensed Matter* **22**, 253202 (2010).

12. Mortensen JJ, Hansen LB, Jacobsen KW. Real-space grid implementation of the projector augmented wave method. *Physical Review B* **71**, 035109 (2005).

13. McLeod AS*, et al.* Model for quantitative tip-enhanced spectroscopy and the extraction of nanoscale-resolved optical constants. *Physical Review B* **90**, 085136 (2014).

14. Wagner M*, et al.* Ultrafast and Nanoscale Plasmonic Phenomena in Exfoliated Graphene Revealed by Infrared Pump–Probe Nanoscopy. *Nano Letters* **14**, 894-900 (2014).

15. Charnukha A*, et al.* Ultrafast nonlocal collective dynamics of Kane plasmon-polaritons in a narrow-gap semiconductor. *Science Advances* **5**, eaau9956 (2019).

16. Johnston DC. The puzzle of high temperature superconductivity in layered iron pnictides and chalcogenides. *Advances in Physics* **59**, 803-1061 (2010).

17. Latini S, Ronca E, De Giovannini U, Hübener H, Rubio A. Cavity Control of Excitons in Two-Dimensional Materials. *Nano Letters* **19**, 3473-3479 (2019).

18. Steinleitner P*, et al.* Direct Observation of Ultrafast Exciton Formation in a Monolayer of WSe2. *Nano Letters* **17**, 1455-1460 (2017).

19. Chernikov A, Ruppert C, Hill HM, Rigosi AF, Heinz TF. Population inversion and giant bandgap renormalization in atomically thin WS2 layers. *Nature Photonics* **9**, 466 (2015).

20. Chernikov A*, et al.* Electrical Tuning of Exciton Binding Energies in Monolayer WS2. *Physical Review Letters* **115**, 126802 (2015).

21. Wang J*, et al.* Optical generation of high carrier densities in 2D semiconductor heterobilayers. *Science Advances* **5**, eaax0145 (2019).

22. Steinhoff A, Rösner M, Jahnke F, Wehling TO, Gies C. Influence of Excited Carriers on the Optical and Electronic Properties of MoS2. *Nano Letters* **14**, 3743-3748 (2014).

23. Schlaus AP*, et al.* How lasing happens in CsPbBr3 perovskite nanowires. *Nature Communications* **10**, 265 (2019).

24. Versteegh MAM, Kuis T, Stoof HTC, Dijkhuis JI. Ultrafast screening and carrier dynamics in ZnO: Theory and experiment. *Physical Review B* **84**, 035207 (2011).

25. Sun D*, et al.* Observation of Rapid Exciton–Exciton Annihilation in Monolayer Molybdenum Disulfide. *Nano Letters* **14**, 5625-5629 (2014).

26. Wang H, Zhang C, Rana F. Ultrafast Dynamics of Defect-Assisted Electron–Hole Recombination in Monolayer MoS2. *Nano Letters* **15**, 339-345 (2015).

27. Yu Y, Yu Y, Huang L, Peng H, Xiong L, Cao L. Giant Gating Tunability of Optical Refractive Index in Transition Metal Dichalcogenide Monolayers. *Nano Letters* **17**, 3613-3618 (2017).

28. Kravets VG, Wu F, Auton GH, Yu T, Imaizumi S, Grigorenko AN. Measurements of electrically tunable refractive index of MoS2 monolayer and its usage in optical modulators. *npj 2D Materials and Applications* **3**, 36 (2019).

29. Sun Z, Martinez A, Wang F. Optical modulators with 2D layered materials. *Nature Photonics* **10**, 227 (2016).

30. Soref R, Bennett B. Electrooptical effects in silicon. *IEEE Journal of Quantum Electronics* **23**, 123-129 (1987).

31. Scuri G*, et al.* Large Excitonic Reflectivity of Monolayer MoSe2 Encapsulated in Hexagonal Boron Nitride. *Physical Review Letters* **120**, 037402 (2018).

32. Haastrup S*, et al.* The Computational 2D Materials Database: high-throughput modeling and discovery of atomically thin crystals. *2D Materials* **5**, 042002 (2018).

33. Liang WY. Optical anisotropy in layer compounds. *Journal of Physics C: Solid State Physics* **6**, 551-565 (1973).
